# Gait patterns during overground and virtual omnidirectional treadmill walking

**DOI:** 10.1186/s12984-023-01286-6

**Published:** 2024-02-22

**Authors:** Morgan McGrath Lewis, Colin Waltz, Logan Scelina, Kathryn Scelina, Kelsey M. Owen, Karissa Hastilow, Eric M. Zimmerman, Anson B. Rosenfeldt, Mandy Miller Koop, Jay L. Alberts

**Affiliations:** 1 Department of Biomedical Engineering, Cleveland Clinic, Cleveland, OH USA; 2 School of Medicine, Case Western Reserve University, Cleveland, OH USA; 3 Neurological Institute, Center for Neurological Restoration, Cleveland Clinic, Cleveland, OH USA

**Keywords:** Gait, Locomotion, Kinematics, Virtual reality, Omnidirectional treadmill

## Abstract

**Background:**

Omnidirectional treadmills (ODTs) offer a promising solution to the virtual reality (VR) locomotion problem, which describes the mismatch between visual and somatosensory information and contributes to VR sickness. However, little is known about how walking on ODTs impacts the biomechanics of gait. This project aimed to compare overground and ODT walking and turning in healthy young adults.

**Methods:**

Fifteen young adults completed forward walk, 180° turn, and 360° turn tasks under three conditions: (1) overground, (2) on the Infinadeck ODT in a virtual environment without a handrail, and (3) on the ODT with a handrail. Kinematic data for all walking trials were gathered using 3D optical motion capture.

**Results:**

Overall, gait speed was slower during ODT walking than overground. When controlling for gait speed, ODT walking resulted in shorter steps and greater variability in step length. There were no significant differences in other spatiotemporal metrics between ODT and overground walking. Turning on the ODT required more steps and slower rotational speeds than overground turns. The addition of the stability handrail to the ODT resulted in decreased gait variability relative to the ODT gait without the handrail.

**Conclusion:**

Walking on an ODT resembles natural gait patterns apart from slower gait speed and shorter step length. Slower walking and shorter step length are likely due to the novelty of physically navigating a virtual environment which may result in a more conservative approach to gait. Future work will evaluate how older adults and those with neurological disease respond to ODT walking.

**Supplementary Information:**

The online version contains supplementary material available at 10.1186/s12984-023-01286-6.

## Background

Over the past two decades, significant advancements in virtual reality (VR) technology have resulted in multiple applications of VR related to medical education [1, 2], surgical planning [2, 3], pain management [4–7], patient education [8], and rehabilitation [9, 10]. However, the utility of VR in medicine has not been fully realized, particularly in the evaluation and treatment of neurological and motor disorders. For example, performance of complex tasks under realistic conditions may reveal subtle deficits in motor control or cognitive functioning that are typically overlooked in traditional clinical tests, thus providing additional insight into disease progression and providing a target for treatment [11–14]. Capturing gait deficits (e.g., freezing of gait in Parkinson’s disease) in a traditional clinical setting is challenging [15, 16]. Virtual reality offers a means to create immersive digital representations of everyday environments and tasks to evaluate motor function in real world contexts. Barriers to using VR for the assessment and potential treatment of neurological populations include the VR locomotion problem (i.e., navigation of a large virtual environment within the confines of a smaller physical space) and an understanding of how gait patterns are impacted while walking in a VR environment.

Sensory inconsistencies between the visual and vestibular systems while completing tasks in traditional VR setups often result in nausea or physical discomfort [17]. Typical approaches to VR navigation include: (1) continuous virtual movement with a controller joystick, (2) non-continuous virtual movement through point-and-click teleportation between locations, and (3) matching the size of the virtual space with the size of the available physical space. These approaches are problematic as they can cause motion sickness from the sensory mismatch between visual flow and vestibular information, break the user’s sense of immersion, critically limit the structure and scale of possible VR environments, and, as in cases 1) and 2), fail to provide any information about gait function [18–21].

Recent VR applications have combined traditional unidirectional treadmills with simple VR environments in which the user controls a virtual avatar during treadmill walking [22–28]. This approach improves immersion and reduces symptoms of motion sickness by fusing virtual and physical movement; however, it necessarily limits the complexity of gait during the VR experience as multi-directional movements and turning cannot be completed [29, 30].

The growth in VR gaming has resulted in commercial availability of omnidirectional treadmills (ODTs) [31–35]. Omnidirectional treadmills utilize various mechanical approaches to allow the user to move more naturally within virtual environments, including low-friction flat surfaces, concave surfaces, and systems of traditional treadmill belts. One such belt-based platform is the Infinadeck ODT (Infinadeck, Rocklin, CA), which is a large treadmill in one axis that carries several smaller treadmills in the perpendicular axis [36, 37]. The treadmill’s motion is user-paced and responds to the direction and acceleration of a VR motion tracker worn by the user [38].

Advances in technology related to the development and control of ODTs may make them a viable approach to addressing the long-standing VR locomotion problem and promote the evaluation of gait under controlled, realistic conditions. Numerous studies have compared overground and traditional treadmill walking [39–43], but to date few studies have systematically evaluated gait kinematics during overground versus ODT locomotion. An evaluation of speed adaptation on the Cyberith Virtualizer ODT, a low friction walking device, indicated that ODT walking is characterized by consistently slower gait speeds, increased cadence, and shorter step lengths when compared to overground forward walking in a virtual environment [44]. Similarly, one study investigating the CyberWalk ODT, a belt-based system, also reported slower speeds, increased cadence, shorter steps lengths, and higher gait variability [45]. Although previous studies have evaluated gait while following curved pathways, a gap remains in understanding how gait on an ODT is affected during turning. Considering the importance of turning and deficits in turning behavior linked to limited mobility and falls in neurological patients, it is necessary to characterize the kinematics of turning during ODT locomotion [46–48].

The limited evidence available suggests ODT locomotion may be impacted by the challenging gait conditions, such as turning, and feelings of instability associated with the novelty of ODT walking combined with the lack of visual perception of the physical environment when using an immersive VR headset. Other factors contributing to differences with overground walking include haptic feedback from the harness systems used with ODTs and differing shear forces between the foot and walking surface when using ODTs with concave or low-friction platforms. Taken together, it is unreasonable to assume that ODT locomotion directly imitates overground gait, and the specific mechanical approach of each ODT device likely plays a role in gait adaptation.

A necessary precursor to the implementation of VR paradigms that utilize ODTs in neurology or rehabilitation is to determine the differences in overground and ODT gait patterns. As ODTs offer a distinct advantage over unidirectional treadmills in the ability to evaluate gait under realistic VR conditions that mimic the complex demands of everyday motor control (e.g., turning and changing directions in response to stimuli), ODT locomotion must be characterized for both simple forward walking and turning tasks. This understanding will facilitate appropriate development of VR environments and interpretation of outcomes, as well as provide a necessary framework for evaluating ODT locomotion in a rehabilitation context. The aim of this project was to use kinematic outcomes to compare overground and ODT walking and turning in a VR environment in healthy young adults. We hypothesized that forward gait on the Infinadeck ODT would be characterized by slower speeds, shorter steps, and increased variability in step length, and ODT turning would be prolonged compared to overground walking.

## Methods

### Participants

Fifteen healthy adults participated in the study (8 males and 7 females, age = 25.1 ± 4.0 years). This sample size is consistent with similar evaluations of the Cyberith Virtualizer ODT [44] and the CyberWalk ODT [45], which each analyzed data from 12 healthy young adults. Demographic data are presented in Table 1. Participants were free from any neurocognitive conditions or musculoskeletal abnormalities that would affect cognitive functioning or gait. The experiment protocol was approved by the Cleveland Clinic human research ethics committee, and all participants completed the informed consent process prior to entering the study.

### Equipment set up and procedure

All study procedures were performed in the Neural Control Laboratory at the Cleveland Clinic, and each data collection session required approximately three hours. In addition to anthropomorphic measurements (height, weight, leg lengths, and joint widths), participants provided demographic details and information on prior experience with VR and ODTs. Each participant was fitted in a full-body harness and three VIVE virtual reality tracking devices (HTC Corporation, Taiwan): one affixed to the lower back via a waist belt and one device affixed to each foot. Participants completed the Simulator Sickness Questionnaire (SSQ) [49] before and after performing all walking trials.

Full body kinematics were captured using a 16-camera Vicon motion capture system (Vicon Motion Systems LTD, UK). Thirty-seven passive retro-reflective markers were placed on specific bony landmarks, as specified by a modified Plug-In-Gait model [50–52]. The Plug-In-Gait model details marker position and is used to transform raw positional data of the markers into kinematic and kinetic gait variables within the Vicon Nexus software. This model was modified for the present study by removing the four head markers, as these would have been occluded by and interfered with the VR headset. The positions of the reflective markers were recorded at a sampling rate of 100 Hz during each overground and treadmill trial. The motion capture system was calibrated prior to each data collection session, and each participant performed one static calibration trial to assign proper anatomical labels to the body markers within the Vicon Nexus software.

### Overground walking trials

**Fig. 1:**
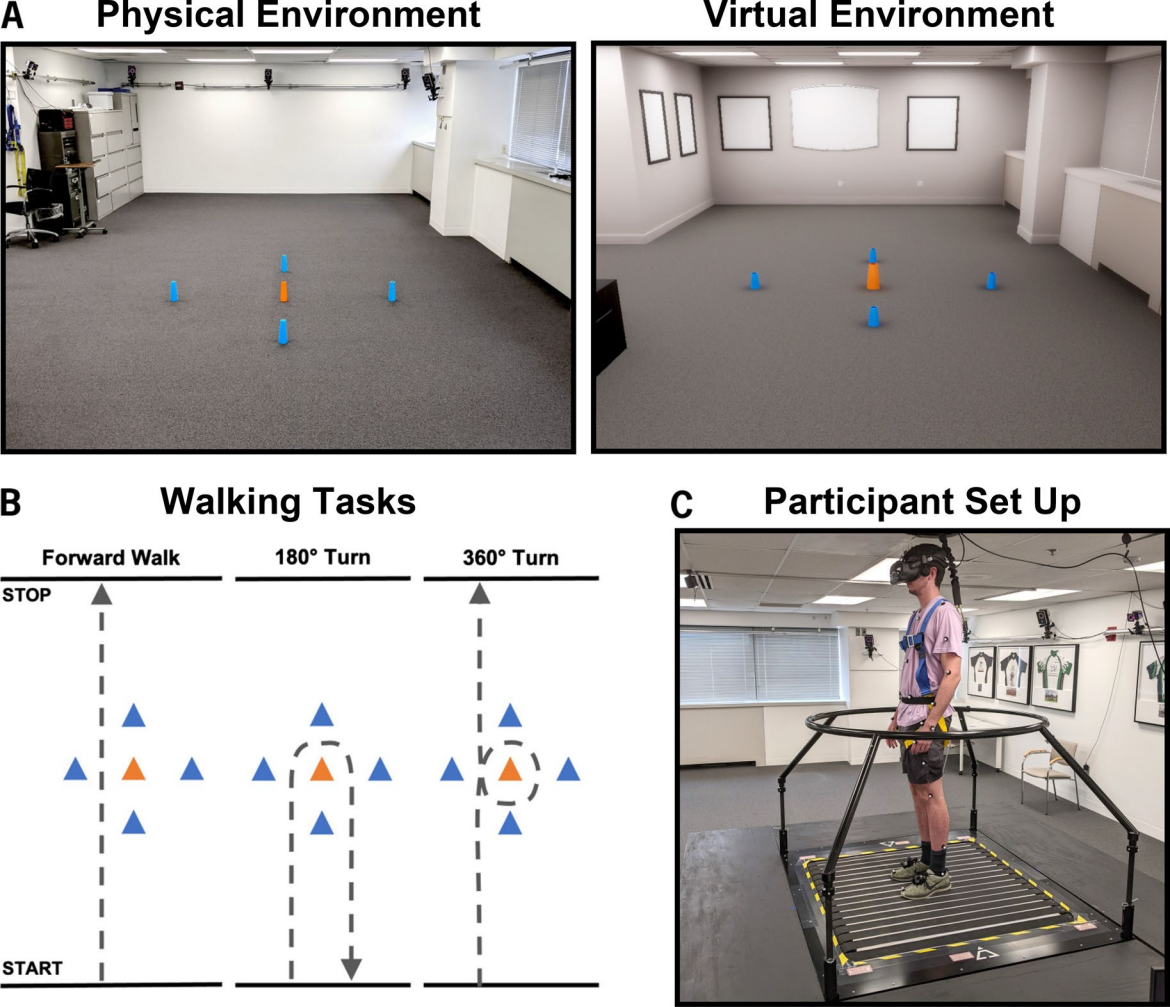
(**A**) Physical and virtual testing environments as viewed from the start position for all trials. (**B**) Diagram of forward walk, 180% turn, and 360% turn tasks to be completed overground and in the ODT + VR. (**C**) Infinadeck ODT with handrail installed, Value Index headset, and ceiling-mounted full-body harness in place. Reflective markers corresponding with Nexus Plug-In-Gait model are visible

The overground environment consisted of a large, open area free of obstacles and distractions. One center cone was placed in the middle of the open area, surrounded by four cones arranged 1 m in the positive and negative X- and Y-directions (Fig. 1A, left). One reflective marker was placed atop each cone to provide context during data analysis. Only kinematic data captured within a designated 4 × 2 m boundary around the center cone were included in analysis, although participants were instructed to start and stop walking outside the bounds of the data capture space to eliminate effects of acceleration and deceleration. Participants were provided a demonstration of each walking task: (1) forward walking, (2) forward walk to righthand 180° turn around center cone, and (3) forward walk to righthand 360° turn around center cone (Fig. 1B). Participants completed two trials of each task at a comfortable walking pace, following verbal start and stop cues for each trial.

### Omnidirectional treadmill + VR walking trials

Steam VR Room Set Up and Infinadeck ODT calibration were performed prior to each session, as previously described [38]. Three stationary base stations (Valve Corporation, Bellevue, WA) were arranged facing the treadmill to monitor the position of the VR headset and VIVE tracking devices throughout treadmill trials. The Infinadeck ODT was chosen for this study due to its similarity to traditional treadmills used for gait analysis as well as the advantage of not requiring any specialized footwear to be worn by the user.

Participants were provided an explanation and demonstration of how to walk and turn on the ODT, followed by time to acclimate to the treadmill prior to donning the Valve Index headset (Valve Corporation, Bellevue, WA). Study staff helped participants adjust the headset to ensure the visual display was clear, and the body harness was secured (Fig. 1 C). The virtual environment replicated the dimensions and design of the physical environment, including the same five-cone arrangement in the center of the space (Fig. 1A, right). Participants were given a habituation period to explore the environment and become comfortable with walking and turning on the treadmill in VR. Habituation was considered complete when the participant reported feeling comfortable and study staff determined the participant had achieved a consistent gait.

Participants repeated the same three tasks (forward walk, 180° turn, 360° turn) as in the overground condition, completing a total of five trials per task. All trials for a task were completed before advancing to the next task. To evaluate the potential impact of a balance aid during ODT walking, following completion of the initial VR trials, a circular handrail attachment was installed around the perimeter of the treadmill platform (Fig. 1 C), and participants completed two additional trials of each task. If a participant reported symptoms of motion sickness, the handrail trials were not performed. Total time in the VR environment was approximately 60 min.

### Data processing

Kinematic data captured within the designated boundaries of the overground motion capture space were included for analysis, representing a linear distance of 4 m in the forward walking direction. Participants started and stopped walking outside of this range to minimize effects of acceleration and deceleration on gait biomechanics. For overground trials, cone reference markers were used to truncate data to the appropriate range. For treadmill trials, kinematic data capture *Start* and *Stop* was automatically triggered based on relative position in the VR space using a custom Raspberry Pi linking the motion capture and VR systems. Thus, kinematic data from both overground and ODT trials represent the same 8m^2^ area in the physical and virtual spaces.

All kinematic data were labeled using a modified Vicon Plug-In-Gait marker set to create a three-dimensional skeleton in the Vicon Nexus software. Small gaps in trajectory data were filled using rigid body techniques in Nexus. Custom MATLAB (R2021a) scripts identified heel strike and toe off events, as well as turn onset and offset events using pelvic rotation. Kinematic data were filtered using 2nd order and 4th order lowpass Butterworth filters prior to identifying foot and turn events, respectively. Heel strikes were identified using whichever came first between minimum vertical heel position and maximum anterior heel position. Toe off events were identified as the maximum posterior distance of the toe marker between heel strikes. Turn segments were identified as the longest consecutive segment where pelvic rotational velocity was > 95% of the mean and the segment covered at least 80% of the rotation needed to complete the given turn task.

Spatiotemporal outcome metrics were calculated by Vicon ProCalc software and custom MATLAB scripts based on foot events and turn identification as described previously. Average gait velocity for forward walk trials was calculated as the total linear distance traveled (4 m) divided by the trial duration. Step length was defined as the 2D anteroposterior distance between the right and left heel at consecutive heel strikes. Step width was defined as the 2D mediolateral distance between the right and left heel at consecutive heel strikes. Cadence was calculated as the frequency of steps within the trial. Step time was defined as the time between consecutive heel strikes; whereas stance time was the time between consecutive heel strike and toe off events, and swing time was the time between consecutive toe off and heel strike events. Double limb support percentage was calculated as the percentage of the gait cycle for which both feet were in contact with the ground. All forward walk metrics were reported as an average per trial as well as the coefficient of variation (standard deviation divided by the mean, multiplied by 100) for the trial. Turn metrics included total turn duration and number of steps in the turn, as determined by the turn segment identified using pelvic rotation data, as well as average and maximum turn velocity, calculated using angular velocity of the pelvis during turning.

### Statistical analysis

The effect of condition (overground, treadmill, treadmill + handrail) on spatiotemporal outcomes was evaluated using linear mixed models (LMMs) fitted by restricted maximum likelihood [53]. Linear mixed models are especially well-suited to handle data with repeated measures but do not require the same number of observations across subjects as do repeated measures ANOVA analyses. In the case of this dataset, there were a number of participants for which specific trials were excluded due to issues in data collection (e.g., stumble steps) and data processing (e.g., missing marker trajectories). Linear mixed models handle this missingness while also accounting for the natural variation in gait patterns between individuals through random effect terms.

Each LMM was fitted with the forward walk outcome of interest as the response variable and included fixed effects of condition and average gait speed during the trial. Models for turn outcomes included only condition as a fixed effect. To account for differences in gait between individuals, models included random intercepts per participant as well as random slopes by condition per participant. All models were validated by visual inspection to confirm residuals and random slopes had nearly normal distributions. In models where the condition term was significant, post-hoc pairwise comparisons were performed using the Kenward-Roger approximation of degrees of freedom and Tukey adjustments for multiple comparisons.

All statistical analyses were performed in RStudio (version 2022.02.3). Models were fitted using the “lme4” package (Bates, Maechler, Bolker, & Walker, 2015) and evaluated using the “lmerTest” package (Kuznetsova, Brockhoff, & Christensen, 2017). Diagnostic plots for LMMs were built using the “redres” package (Goode, McClernon, Zhao, Zhang, & Huo, 2019). Post-hoc pairwise comparisons used the “emmeans” package (Lenth, 2022) to compute contrasts with Tukey adjustments. Statistical significance was set at 0.05 for all tests.

## Results

### Participants

While 40% of participants reported having any prior experience with immersive virtual reality, only four (27%) had used immersive VR within the past year and only one (7%) used immersive VR regularly (Table 1). No participant reported any prior experience with an ODT.

**Table 1:** Participant demographics (N = 15)

Age (years), range 18–30	25.1 ± 4.0
Female	7 (47%)
Height (m)	1.74 ± 0.11
Weight (kg)	76.6 ± 21.3
BMI	24.9 ± 5.0
Any prior immersive VR experience	6 (40%)
Frequency of VR use in the past year	
Never	11 (73%)
Between one and five times	3 (20%)
Greater than five times	1 (7%)
Any prior omnidirectional treadmill experience	0 (0%)

Data presented as mean ± standard deviation or number (percentage)

### Trials included in analysis

Three participants did not complete all trials in the treadmill + handrail condition due to motion sickness. Forty trials were excluded due to missteps or loss of balance, and 24 trials were excluded due to miscellaneous software malfunctions during data collection. In sum, 326 trials across all tasks and conditions were included in the analysis.

### Adjusted model analysis

Intermediate analyses constructed fully adjusted LMMs that included fixed effects of sex, leg length, and BMI, in addition to condition and speed. Adjusted model summaries are presented in Tables S1 – S3. These additional covariates had no meaningful impact on the effects of condition or speed in any model, and all presented findings result from the models specified in Methods.

### Forward walking

#### Impact of gait speed

Participants were instructed to complete all trials at a comfortable pace. For all forward walk trials, average gait speed was significantly faster overground (1.3 ± 0.18 m/s) compared to treadmill (0.43 ± 0.07 m/s, *p* < 0.001) and treadmill + handrail (0.50 ± 0.04 m/s, *p* < 0.001) conditions. Based on the results of forward walk LMMs, gait speed had a significant effect on every outcome, except step width (Table 2) (*p* < 0.001 to 0.015).

**Table 2:** Model estimates of the impact of velocity on spatiotemporal outcomes

	Velocity (m/s)		
**Outcome Measure**	**Estimate (95% CI)**	**SE**	***p*** value
Step Length (m)	0.34 (0.27, 0.40)	0.03	**< 0.001**
Step Width (m)	-0.01 (-0.06, 0.04)	0.02	0.713
Cadence (steps/min)	39.7 (27.7, 52.0)	5.92	**< 0.001**
Step Time (s)	-0.20 (-0.27, -0.13)	0.03	**< 0.001**
Stance Time (s)	-0.35 (-0.47, -0.23)	0.06	**< 0.001**
Swing Time (s)	-0.05 (-0.08, -0.01)	0.02	**0.015**
Double Limb Support (%)	-16.7 (-22.0, -11.8)	2.11	**< 0.001**

Estimate represents the change in the outcome for each 1 m/s increase in gait speed, assuming all other variables are held constant, as predicted by the linear mixed model

CI = confidence interval, SE = standard error

**Fig. 2:**
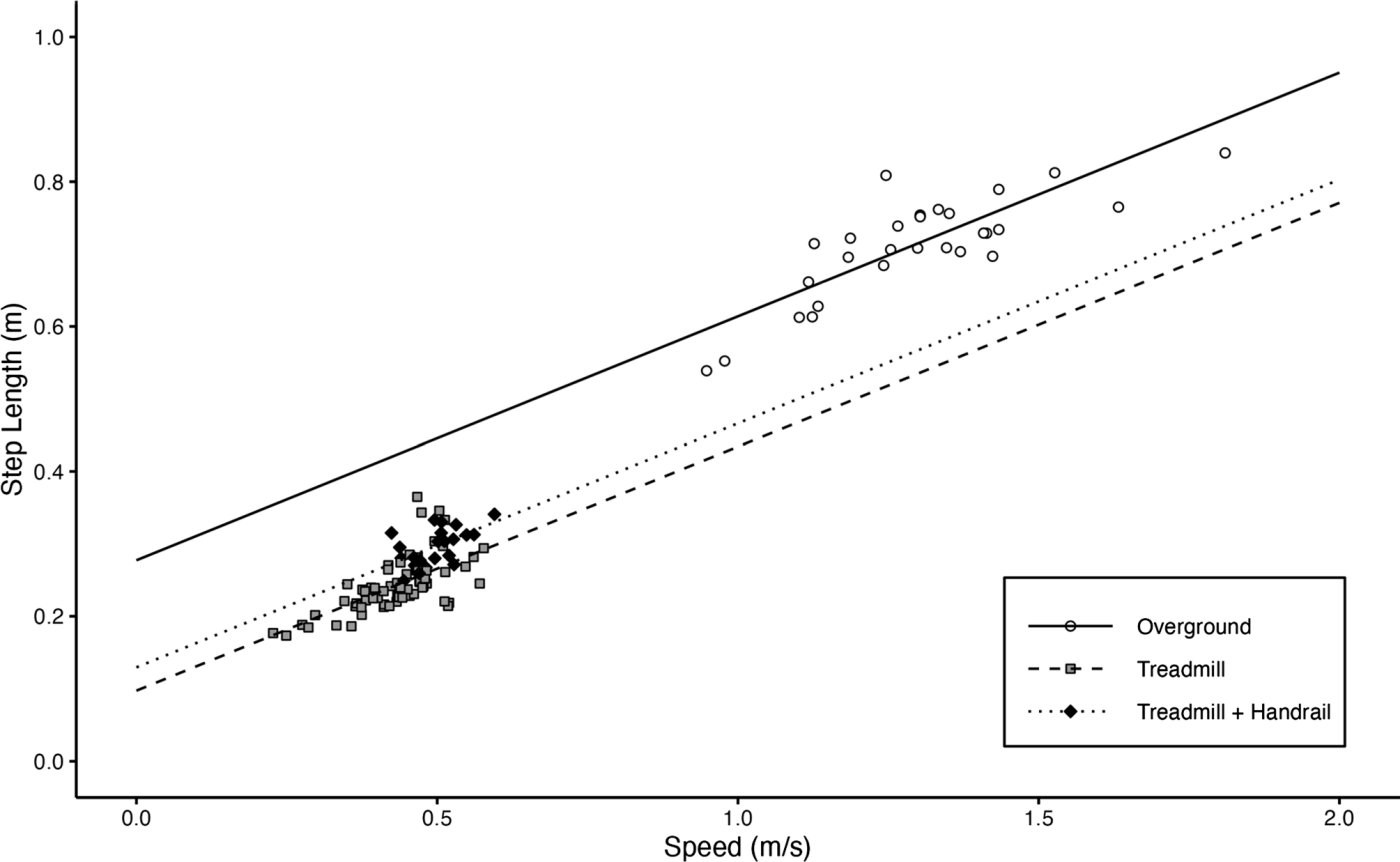
Average step length for each forward walk trial is plotted versus average gait speed for the trial, with the corresponding linear mixed model (LMM) overlaid for each condition. The LMM includes fixed effects of condition and velocity, as well as random intercepts and slopes by condition for participant ID. Each point represents the average step length for a single forward walk trial. The distribution of the step length data, as well as the LMM fit, are representative of all spatiotemporal outcomes and models

Figure 2 depicts results for a single spatiotemporal outcome to provide a representative visualization of both raw data distribution and model fit for the three walking conditions. In particular, data from the overground condition span a larger range of gait speeds and display a clear linear relationship with speed, whereas data from both treadmill conditions are tightly clustered within a narrow range of gait speeds. By including gait speed as a fixed effect in the models, post-hoc pairwise comparisons between conditions test for significant differences between model estimates for the three conditions at a given gait speed.

### Impact of walking condition

**Fig. 3:**
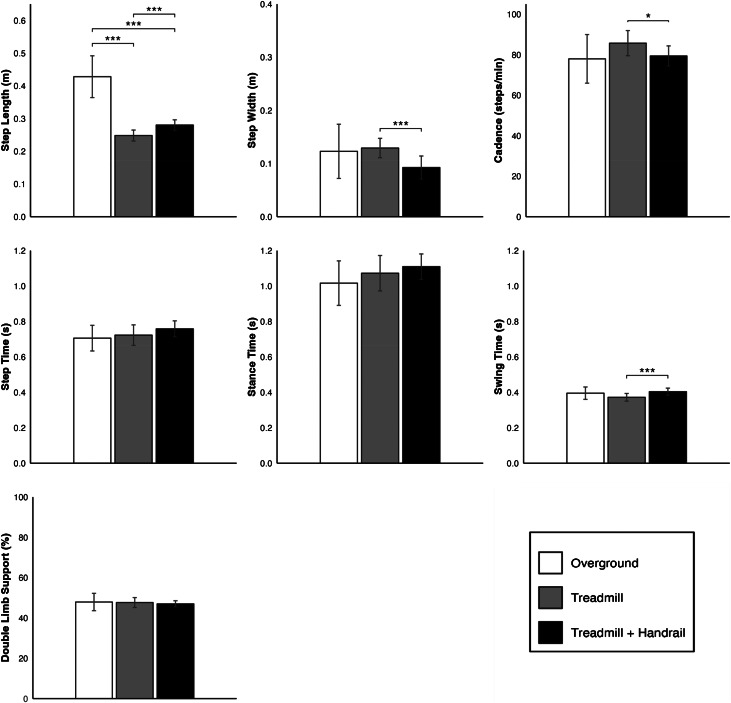
Forward walk model estimates (± 95% confidence intervals) of spatiotemporal outcome metrics when gait speed = 0.45 m/s (the average velocity of all forward walk trials on the treadmill). For models in which the main effect of Condition was significant, post-hoc pairwise comparisons between conditions are illustrated. *p < 0.05, **p < 0.01, ***p < 0.001

Table 3 provides observed values of spatiotemporal outcomes from all forward walking trials, without adjusting for gait speed. After controlling for gait speed using LMMs (Fig. 3), the only significant difference in spatiotemporal outcomes between the overground and treadmill conditions was shorter step lengths for the treadmill + handrail (*p* < 0.001) and treadmill (*p* < 0.001) conditions compared to overground walking. At a gait speed of 0.45 m/s (the average speed of all forward walk trials on the ODT), predicted step lengths for the treadmill, treadmill + handrail, and overground conditions were 0.25 m, 0.28 m, and 0.43 m, respectively. No significant differences were observed in cadence, step width, step time, stance time, swing time, or percentage of the gait cycle spent in double limb support after controlling for speed. When comparing the two treadmill conditions, the addition of the handrail resulted in longer step lengths (*p* < 0.001), narrower step widths (*p* < 0.001), longer swing times (*p* < 0.001), and lower cadence (*p* = 0.037).

To evaluate variability in gait (Table 3), the percent coefficient of variation (CV%) for each outcome was calculated for every trial. The same LMM procedure was used to control for speed and compare CV% across conditions. Gait speed was not a significant predictor for any CV% model (*p* = 0.114 to 0.694). Compared to overground, treadmill walking without the handrail resulted in greater variability in step length (*p* < 0.001) and double limb support (*p* = 0.007). Greater variability in step length (*p* < 0.001) was observed during the treadmill + handrail condition as well. Between the treadmill conditions, the handrail reduced variability in the following outcomes: cadence (*p* = 0.005), step time (*p* = 0.003), stance time (*p* < 0.001), swing time (*p =* 0.001), and double limb support (*p* < 0.001).

**Table 3:** Spatiotemporal outcome metrics for forward walk trials and pairwise comparisons from corresponding linear mixed **models**

				Pairwise Comparisons After Controlling for Velocity		
**Outcome Measure**	**Overground**	**Treadmill**	**Treadmill + Handrail**	Overground – TM	Overground – TM + H	TM – TM + H
Average Velocity (m/s)	1.30 ± 0.18	0.43 ± 0.07	0.50 ± 0.04			
Step Length (m)	0.71 ± 0.07	0.24 ± 0.04	0.29 ± 0.04	**0.18 (0.10, 0.26)**	**0.15 (0.07, 0.22)**	**-0.03 (-0.05, -0.02)**
Step Width (m)	0.12 ± 0.04	0.13 ± 0.03	0.10 ± 0.04	-0.01 (-0.07, 0.05)	0.03 (-0.02, 0.08)	**0.04 (0.02, 0.05)**
Cadence (steps/min)	112 ± 8.5	85 ± 12.2	81 ± 7.3	-7.75 (-23.62, 8.11)	-1.43 (-15.91, 13.04)	**6.32 (0.40, 12.24)**
Step Time (s)	0.54 ± 0.04	0.73 ± 0.11	0.75 ± 0.07	--	--	--
Stance Time (s)	0.72 ± 0.06	1.08 ± 0.19	1.09 ± 0.11	--	--	--
Swing Time (s)	0.36 ± 0.02	0.37 ± 0.04	0.40 ± 0.03	0.02 (-0.02, 0.07)	0.01 (-0.05, 0.03)	**-0.03 (-0.05, -0.01)**
Double Limb Support (%)	33.8 ± 0.01	47.9 ± 0.01	46.3 ± 0.01	--	--	--
CV% Step Length	2.4 ± 1.4	30.9 ± 2.9	32.1 ± 2.2	**-26.4 (-31.8, -21.1)**	**-28.1 (-33.0, -23.2)**	-1.7 (-3.9, 0.6)
CV% Step Width	23.8 ± 9.7	19.3 ± 8.4	15.5 ± 9.0	--	--	--
CV% Cadence	2.2 ± 1.0	8.4 ± 2.5	5.1 ± 1.7	-3.6 (-9.1, 1.8)	-0.5 (-5.5, 4.5)	**3.1 (1.0, 5.3)**
CV% Step Time	2.2 ± 1.0	8.6 ± 2.7	5.2 ± 1.7	-3.3 (-9.2, 2.5)	0.0 (-5.4, 5.4)	**3.3 (1.2, 5.4)**
CV% Stance Time	1.5 ± 0.6	7.2 ± 2.1	4.1 ± 1.5	-3.8 (-8.2, 0.6)	-0.6 (-4.7, 3.4)	**3.1 (1.7, 4.6)**
CV% Swing Time	2.3 ± 1.0	7.7 ± 1.9	5.5 ± 1.1	-3.7 (-8.0, 0.5)	-1.2 (-5.0, 3.7)	**2.6 (1.1, 4.1)**
CV% Double Limb Support	1.8 ± 0.7	5.7 ± 1.1	3.1 ± 0.6	**-4.2 (-7.3, -1.1)**	-1.6 (-4.5, 1.3)	**2.6 (1.6, 3.6)**

Observed data reported as mean ± SD. Pairwise comparisons were calculated only for models in which condition was a significant main effect. Significant (*p* < 0.05) contrast estimates and their 95% confidence intervals are bolded. TM = treadmill, H = handrail, CV% = coefficient of variation as a percentage

### Turning

**Fig. 4:**
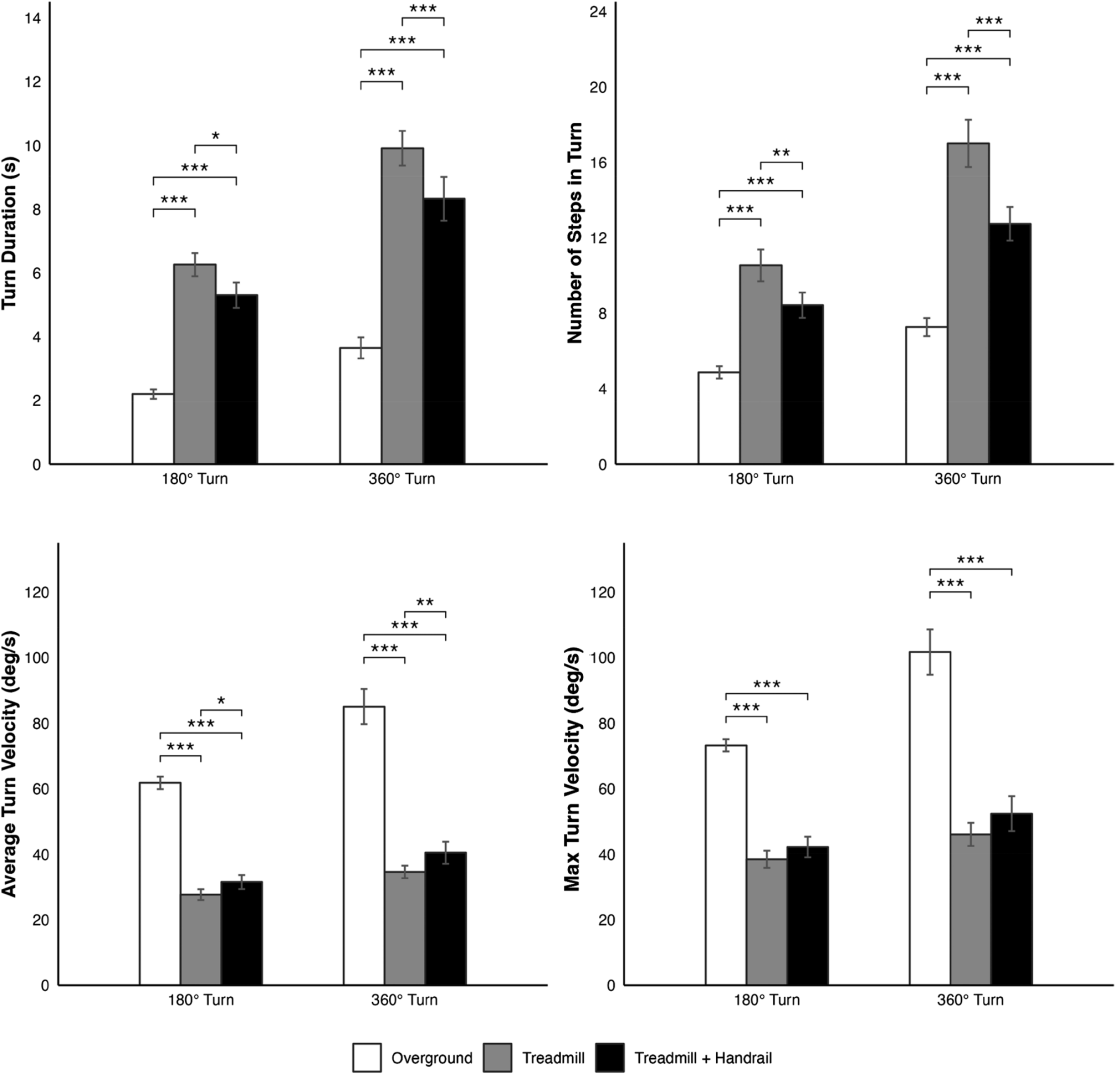
180° and 360° turn model estimates (± 95% confidence intervals) of turning outcome metrics. The main effect of Condition was significant in all models. Statistically significant post-hoc pairwise comparisons are illustrated. *p < 0.05, **p < 0.01, ***p < 0.001

The LMMs for turn outcomes included only condition (not speed) as a fixed effect and participant ID as a random effect. Condition was a significant predictor in all models (*p* < 0.001). Results were consistent across both 180° and 360° turns (Fig. 4; Table 4). Compared to overground turning, turns on the treadmill were longer in duration (*p* < 0.001), required more steps (*p* < 0.001), and had slower average and maximum turning velocities (*p* < 0.001). Between the two treadmill conditions, the addition of the handrail brought treadmill turns closer to natural overground turning in all domains except maximum turn velocity. Compared to those without the handrail, turns with the handrail were of shorter duration (180°: *p* = 0.017, 360°: *p* < 0.001), contained fewer steps (180°: *p* = 0.008, 360°: *p* < 0.001), and had higher average turn velocites (180°: *p* = 0.043, 360°: *p* < 0.008).

**Table 4:** Spatiotemporal outcome metrics for turn trials and pairwise comparisons from corresponding linear mixed models

				Pairwise Comparisons		
**Outcome Measure**	**Overground**	**Treadmill**	**Treadmill + Handrail**	Overground – TM	Overground – TM + H	TM – TM + H
180° Turns						
Duration (s)	2.2 ± 0.2	6.3 ± 0.7	5.3 ± 0.7	**-4.1 (-4.5, -3.6)**	**-3.1 (-3.6, -2.6)**	**1.0 (0.2, 3.2)**
Number of Steps	4.9 ± 0.4	10.6 ± 1.5	8.4 ± 1.2	**-5.7 (-6.7, -4.7)**	**-3.6 (-4.4, -2.7)**	**2.1 (0.6, 3.6)**
Average velocity (deg/s)	61.8 ± 3.5	27.7 ± 3.0	31.6 ± 3.7	**34.1 (31.4, 36.8)**	**30.3 (26.8, 33.7)**	**-3.9 (-7.6, -0.1)**
Max velocity (deg/s)	73.3 ± 3.4	38.4 ± 4.7	42.4 ± 5.3	**34.8 (30.9, 38.6)**	**31.0 (26.8, 35.3)**	-3.8 (-8.4, 0.9)
360° Turns						
Duration (s)	3.7 ± 0.5	9.9 ± 1.0	8.3 ± 1.2	**-6.3 (-6.9, -5.6)**	**-4.7 (-5.4, -4.0)**	**1.6 (0.7, 2.4)**
Number of Steps	7.3 ± 0.8	16.9 ± 2.3	12.8 ± 1.5	**-9.7 (-11.4, -8.1)**	**-5.5 (-6.6, -4.4)**	**4.3 (2.8, 5.7)**
Average velocity (deg/s)	85.0 ± 9.7	34.6 ± 3.4	40.6 ± 5.9	**50.4 (44.1, 56.7)**	**44.6 (39.5, 49.6)**	**-5.9 (-9.8, -1.9)**
Max velocity (deg/s)	101.7 ± 12.5	46.1 ± 3.7	52.6 ± 9.3	**55.6 (46.8, 64.5)**	**49.3 (42.9, 55.7)**	-6.3 (-12.8, 0.2)

Observed data reported as mean ± SD. Pairwise comparisons were calculated only for models in which condition was a significant main effect. Significant (*p* < 0.05) contrast estimates and their 95% confidence intervals are bolded. TM = treadmill, H = handrail, CV% = coefficient of variation as a percentage

### User experience

All participants completed the Simulator Sickness Questionnaire (SSQ) before and immediately after using the ODT (Table 5). In addition to a total score, the SSQ produces sub-scores for nausea, oculomotor, and disorientation symptoms of simulator sickness. Overall, the most common symptoms reported after completion of the ODT trials were sweating (N = 8) and nausea (N = 4), with the majority of participants reporting no to mild symptoms (median total score = 3.7 points out of 235.6 maximum).

**Table 5:** Results of Simulator Sickness Questionnaire before and after treadmill walking trials

	Pre	Post	*p* value
SSQ Total (max = 235.6)	0.0 [0.0, 5.6]	3.7 [1.9, 24.3]	**0.044**
Nausea Sub-score (max = 200.3)	0.0 [0.0, 9.5]	9.5 [0.0, 33.4]	**0.024**
Oculomotor Sub-score (max = 159.2)	0.0 [0.0, 7.6]	7.6 [0.0, 11.4]	0.223
Disorientation Sub-score (max = 292.3)	0.0 [0.0, 0.0]	0.0 [0.0, 27.8]	**0.027**

Data reported as median [Q1, Q3]. Pre and Post scores compared using one-sided paired Wilcoxon Signed Rank test

## Discussion

Omnidirectional treadmills offer a promising solution to the VR locomotion problem by allowing users to physically navigate large, realistic virtual environments to reflect actions in the real world without, in general, substantial VR sickness as captured by the SSQ. To realize the potential in combining VR content and ODT technology, it is necessary to understand the possible differences and similarities between typical overground and ODT walking in VR. Based on results of LMMs assessing spatiotemporal gait metrics across walking conditions, the gait pattern of ODT walking was not significantly different than overground walking for healthy young adults, with a few notable exceptions.

### Kinematic changes to gait during ODT walking

Gait speed was approximately 65% slower during ODT walking compared to overground, despite instruction to perform all trials at a comfortable pace. This is consistent with existing evidence from self-paced walking in VR environments while on a unidirectional treadmill. Previous studies have reported slower self-selected walking speeds in VR, as well as other indications of a conservative or cautious gait (e.g., increased gait variability, increased step width) [54–57]. A more conservative approach to gait may be due to a sense of isolation from the physical world induced by the fully immersive VR environment presented through a head mounted display (HMD) [56, 58]. However, an evaluation of the much larger CyberWalk ODT indicated consistently slower walking speeds on the ODT compared to overground when wearing a HMD device in both conditions, suggesting the ODT impacted gait beyond VR [45]. Slower walking on ODTs appears to be a general adaptation as even after providing participants with a 15-minute acclimation period, gait speed was slower than overground walking [53]. The habituation period employed in the current study most likely provided sufficient familiarization; there was no difference in gait speed as a function of trial number. Taken together, this evidence suggests slower gait speed is a fundamental characteristic of ODT walking.

Omnidirectional treadmill walking is slower than overground walking even after attempts to standardize pace across conditions. Soni and Lamontagne aimed to characterize speed adaptation on a different omnidirectional locomotion system, the Cyberith Virtualizer, and reported the walking speeds achieved in the “fast” ODT condition were comparable to those achieved in the “slow” overground condition in a group of healthy young adults [44]. Both with and without a VR environment presented through a HMD, gait speed was approximately 0.65 m/s slower on the treadmill than overground. Therefore, should clinical applications use VR + ODT paradigms, walking speed should not be directly related or compared to overground walking in terms of characterizing community ambulation capability [59].

Very little work has been done to characterize overground gait in healthy adults at very slow gait speeds (< 0.5 m/s). However, the impact of speed on spatiotemporal gait metrics is well-established [60–63]. These relationships were replicated in the present findings, as gait speed was a significant predictor of every forward walk outcome except step width (Table 2). Across all conditions, faster gait speed was associated with longer step lengths, higher cadence, shorter step times, shorter swing and stance times, and less time spent in double limb support. Gait speed was not associated with any change in the variability (CV%) of any spatiotemporal metric. The relationships between gait speed and other spatiotemporal gait metrics during ODT locomotion are consistent with those established for overground walking, suggesting that ODT walking mimics natural gait patterns [64]. These relationships should continue to be evaluated as ODT hardware and software improve and gait speed becomes more reflective of overground walking.

By including speed as a fixed effect in the LMMs, it was possible to compare model estimates for spatiotemporal outcomes across the three conditions at any given gait speed. To produce model estimates with reasonable uncertainty and avoid extrapolating beyond the range of the observed data, Fig. 3 depicts model estimates across conditions for every outcome when gait speed is 0.45 m/s, the average gait speed of all forward walk trials on the treadmill. The models predict that overground and ODT walking are not significantly different with respect to cadence, step width, step time, stance and swing time, nor double limb support. However, step length is shorter on the ODT (0.25 m) than it is predicted to be overground (0.43 m) at slow speeds. Souman et al. similarly reported shorter steps on the CyberWalk ODT compared to overground, although gait parameters were calculated using head position, and gait speed was not controlled for across conditions [45].

Even after accounting for gait speed, the short steps on the Infinadeck ODT may be the result of limited dimensions of the walking platform. Compared to the CyberWalk ODT, which has a walking surface of 4 m x 4 m, the Infinadeck platform is approximately 1.2 m x 1.2 m. Users walk at the outer boundary of the platform to bring the waist-worn tracker to the periphery of the walking surface, thus achieving the highest speed possible on the treadmill but limiting maximum step length. Notably, the addition of the stability handrail provided somatosensory feedback about body position in space relative to the edge of the walking platform, allowing participants to marginally increase both gait speed (16%) and step length (13%) relative to ODT walking without the handrail.

In addition to shorter steps, forward walking on the treadmill was also characterized by greater variability in step length. This was observed both with and without the handrail and indicates a deviation from a natural gait pattern [65]. Participants were unable to walk at their preferred step length, and instead relied on feedback from the treadmill speed and the feeling of the surface underfoot to inform necessary modulations in step length from one gait cycle to the next. Treadmill walking without the handrail also involved greater variability in double limb support time. It has been hypothesized that step variability may reflect an effort to preserve mechanical stability when walking [65]. This hypothesis is supported by the present findings of greater CV% for all spatiotemporal outcomes (except step length) on the treadmill compared to the treadmill + handrail condition. Despite the utilization of a full-body harness for all ODT trials and the absence of any falls or near-falls during ODT walking, participants reported increased levels of comfort with the handrail installed. Not only did the handrail offer balance support, but it also provided tactile feedback to keep participants oriented in the physical space while navigating in VR.

### Potential mechanisms underlying altered ODT walking

There are likely several biological mechanisms at play that influence the distinct deviations from normal gait (slow speed, short steps, greater variability in step length) observed in ODT locomotion. First, the use of a HMD and VR environment, which obstructs the view of the physical environment, introduces a higher degree of task complexity for even basic forward walking tasks performed on this system compared to overground. In both healthy adults and those with neurological disease, gait speed and gait variability are negatively affected by dual tasking, and the effect is magnified with increasing dual task load [66, 67]. Second, the HMD and perceived instability on the treadmill may induce a fear of falling, which is also associated with similar spatiotemporal changes in gait: slower speed, shorter steps, and increased variability [68, 69]. The gait patterns observed in the treadmill condition resemble high-complexity dual tasking and fear of falling. The added stability of the treadmill + handrail condition reduced gait variability and increased speed, step length, and cadence. This supports the use of the handrail in future applications of this system to reduce the influence of instability and fear of falling on gait.

Beyond gait speed and step length, there were no differences in other spatiotemporal gait outcomes between overground and treadmill walking despite the novelty of the ODT + VR system for all participants. After a brief habituation period (< 5 min for all participants), gait cycles largely resembled natural walking in terms of stance, swing, and double limb support phases. The consistency of gait in a unique environment and on a complicated walking surface points to the importance of both stability and flexibility in the neural control of walking. Variability, while traditionally viewed as evidence of a detriment in motor control [70, 71], is an essential compensatory mechanism that maintains critical gait patterns in the face of novel stimuli and disturbances in normal walking [72]. However, these healthy compensatory strategies deteriorate with aging and neurological disease, reducing adaptability and contributing to increased fall risk and fear of falling [68, 70, 73]. Omnidirectional treadmill + VR systems offer a safe and controlled way to introduce motor and cognitive challenges to evaluate gait in complex situations.

### Turning behavior during ODT walking

This is the first study to systematically evaluate turning on an ODT compared to overground. As with forward walking, all turns on the treadmill were performed at slower speeds than overground. The duration and number of steps in treadmill turns were two to three times higher than overground turns. Turns performed with the handrail on the treadmill were overall faster and required fewer steps than those without the handrail, but they were still significantly different than overground turns. Despite these differences in traditional turning metrics, this ODT system facilitates more natural turn navigation than other systems that utilize a stationary waist harness and only allow stabilized rotation. On the Infinadeck, users turn by ambulating around the outer boundary of the walking platform, replicating the path followed when turning around an overground cone. Similar to limitations during forward walking, gait while turning is likely affected by the increased complexity of the motor task and perceived fear of falling.

The potential to quantify turning behavior in realistic complex environments is a major benefit of ODT systems. Importantly, turning is an essential daily activity required for many basic functional tasks, and turn steps make up approximately 35–40% of all steps in a typical day [74]. Aging [75] and neurological disease [76, 77] are both associated with deficits in efficiency and quality of turning, resulting in increased fall risk [78]. In particular, freezing of gait (FoG) in Parkinson’s disease (PD) can be triggered by complex scenarios such as turning while in tight spaces or performing a simultaneous cognitive task [79, 80]. The episodic and multifactorial nature of FoG makes it difficult to capture in traditional clinical settings, but FoG has important implications for overall quality of life, medication management, and mitigation of fall risk in PD [81–83]. With a VR + ODT system, it is possible to recreate everyday scenarios that commonly trigger FoG episodes and capture detailed biomechanical data surrounding the phenomenon.

Omnidirectional treadmill systems offer promising opportunities to improve the evaluation and management of neurological and motor disorders such as PD. It is essential, therefore, that the systems are well-tolerated in terms of VR sickness. A recent meta-analysis reported that while having a neurological disorder is positively correlated with experiencing VR sickness, age does not have a significant relationship with VR sickness [84]. Other data indicate older adults and individuals with PD experience low levels of motion sickness following exposure to simple VR environments [23], but none of these studies have combined VR with an ODT. Fusing the physical and virtual worlds by combining VR visual flow with natural locomotion will ultimately reduce the sensory mismatch of traditional VR movement paradigms and reduce the experience of motion sickness symptoms [19, 85].

### Minimal cybersickness during ODT walking

Although the present study included only healthy young adults, results from the SSQ revealed the majority of participants experienced no symptoms (4 of 15, 27%) or mild symptoms (8 of 15, 53%) of VR sickness after approximately an hour of walking and turning on the treadmill. Three participants were unable to complete the total number of treadmill + handrail trials due to feelings of motion sickness and subsequently reported higher scores on the SSQ. The present protocol involved a total of 14 turns around virtual cones, in addition to turns performed during the habituation period and considerable stationary turning necessary to reorient at the start of each trial. Virtual applications that simulate everyday activities will likely involve purpose-driven turning rather than repetitive prescribed turns around a cone, reducing experience of disorientation symptoms. Sixty total minutes in a VR environment is also a substantial amount of time; our recently developed paradigm to evaluate virtual instrumental activities of daily living is substantially shorter and to date has been well tolerated [38]. Importantly, the SSQ is only one possible tool to quantify symptoms of VR sickness, and its direct applicability to VR systems has been questioned [86]. Additional work is necessary to evaluate VR sickness among various populations, identify specific triggers of sickness within VR applications, and validate VR-specific sickness questionnaires.

#### Limitations

This study was the first to quantify gait parameters using the Infinadeck ODT. As such, the goal was to compare natural overground gait with gait on the treadmill system, and gait speed was not dictated a priori. Although LMMs controlled for gait speed, it is possible that the relationship between gait speed and various spatiotemporal metrics differs between overground walking and ODT walking. Additional work is needed to evaluate ODT locomotion across a wide range of gait speeds.

The sample of young participants with no cognitive or motor impairments offers an opportunity to assess the influence of the ODT + VR system on functional, healthy gait. A sample of healthy older adults and individuals with PD also completed this protocol, and those data are currently being analyzed to understand how aging and pathology impact ODT walking. In addition, the single-session nature of this testing paradigm presents a limitation regarding the translation of these results to ODT applications for rehabilitation purposes, in which patients would generally undergo longer and repeated testing sessions. Additional work is needed to investigate the potential for gait parameters to normalize or be modified over multiple exposures to ODT walking, although the present analysis revealed that trial number within the single testing session had no significant effect on any spatiotemporal gait outcome, indicating the familiarization period provided sufficient exposure to acclimate to the novelty of the ODT. Finally, the hardware and software set-up was unique to this study, and caution should be exercised in extending these results to all ODTs. Gait strategies may differ between belt-based and low-friction ODT systems. To this end, future directions for this work include a direct comparison between a belt-based device and a low-friction device in order to systematically evaluate differences in walking mechanics and user preference between the systems.

## Conclusion

Omnidirectional treadmills can be combined with VR to allow users to physically navigate a large and realistic virtual space. The present study quantified a relationship between overground gait and ODT walking and turning. Results revealed that treadmill walking involved slower gait speed, shorter steps, and more variability compared to overground walking. Similarly, turns were performed more slowly and required more steps. The compromised visual and vestibular feedback due to the immersive VR headset and novelty of the walking platform contributed to gait patterns indicative of decreased stability. Overall, participants reported low levels of VR sickness symptoms following completion of the walking protocol, despite the high number of turns required.

Omnidirectional treadmills show promise as a solution to the VR locomotion problem. They provide opportunity to recreate complex everyday scenarios to better understand and monitor gait deficits in those with neurological function. These systems also have potential to serve as rehabilitation platforms to train gait in real world environments under dual-task conditions.

### Electronic supplementary material

Below is the link to the electronic supplementary material.

**Supplementary Material 1: Table S1.** Adjusted and unadjusted model estimates of the effects of condition, velocity, leg length, BMI, and sex on forward walking outcomes. **Table S2.** Adjusted and unadjusted model estimates of the effects of condition, velocity, leg length, BMI, and sex on variabilityoutcomes. **Table S3.** Adjusted models estimates of the effects of condition, velocity, leg length, BMI, and sex on turn outcomes

## Data Availability

The datasets collected and analyzed during the current study are available from the corresponding author on reasonable request.
