# Supplementary material for: Gait patterns during overground and virtual omnidirectional treadmill walking
Source: J Neuroeng Rehabil. 2024 Feb 22;21:29. doi: 10.1186/s12984-023-01286-6 (PMC10885397; doi:10.1186/s12984-023-01286-6)
Supplement: Supplementary file 1 [file 12984_2023_1286_MOESM1_ESM.docx]

Table S1. Adjusted and unadjusted model estimates of the effects of condition, velocity, leg length, BMI, and sex on forward walking outcomes.

		Condition		Velocity	Leg Length	BMI	Sex
**Outcome Measure**	**Model Type**	Treadmill	Treadmill + Handrail				Female
Step Length (m)	Adjusted	**-0.174 (-0.238, -0.117)**	**-0.143 (-0.203, -0.090)**	**0.343 (0.274, 0.405)**	0.160 (-0.133, 0.467)	-0.001 (-0.004, 0.002)	.002 (-0.043, 0.044)
	Unadjusted	**-0.180 (-0.241, -0.119)**	**-0.148 (-0.204, -0.091)**	**0.337 (0.271, 0.402)**			
Step Width (m)	Adjusted	0.007 (-0.039, 0.054)	-0.030 (-0.070, 0.012)	-0.008 (-0.057, 0.043)	0.298 (0.017, 0.585)	0.003 (-0.000, 0.007)	0.043 (0.003, 0.083)
	Unadjusted	0.006 (-0.039, 0.053)	-0.030 (-0.071, 0.011)	-0.009 (-0.058, 0.042)			
Cadence (steps/min)	Adjusted	2.297 (-8.513, 14.523)	-2.565 (-12.722, 7.853)	**34.105 (22.408, 46.066)**	-37.478 (-82.623, 6.562)	0.312 (-0.187, 0.802)	0.913 (-5.061, 7.236)
	Unadjusted	7.752 (-3.860, 19.773)	1.434 (-8.975, 12.399)	**39.663 (27.690, 52.007)**			
Step Time (s)	Adjusted	0.043 (-0.044, 0.127)	**0.075 (-0.000, 0.147)**	**-0.165 (-0.246, -0.088)**	0.119 (-0.157, 0.399)	-0.001 (-0.004, 0.002)	-0.011 (-0.049, 0.028)
	Unadjusted	0.017 (-0.063, 0.096)	0.052 (-0.017, 0.120)	**-0.196 (-0.266, -0.126)**			
Stance Time (s)	Adjusted	0.097 (-0.056, 0.242)	**0.128 (-0.000, 0.249)**	**-0.298 (-0.439, -0.166)**	0.190 (-0.274, 0.663)	-0.001 (-0.006, 0.004)	-0.014 (-0.078, 0.050)
	Unadjusted	0.056 (-0.083, 0.194)	0.093 (-0.023, 0.209)	**-0.346 (-0.467, -0.225)**			
Swing Time (s)	Adjusted	-0.012 (-0.047, 0.023)	0.019 (-0.012, 0.050)	-0.031 (-0.066, 0.002)	0.069 (-0.068, 0.210)	-0.001 (-0.003, 0.000)	-0.006 (-0.023, 0.012)
	Unadjusted	-0.023 (-0.058, 0.011)	0.008 (-0.022, 0.039)	**-0.045 (-0.078, -0.012)**			
Double Limb Support (%)	Adjusted	1.592 (-2.650, 6.169)	0.642 (-3.075, 4.346)	**-14.512 (-18.781, -10.184)**	10.954 (0.269, 21.710)	0.108 (-0.012, 0.217)	0.170 (-1.594, 1.681)
	Unadjusted	-0.263 (-5.446, 4.640)	-0.922 (-5.467, 3.138)	**-16.659 (-22.004, -11.802)**			

Results reported as model estimate and 95% confidence interval. Significant terms are shown in bold ($p < 0.05$). Reference categories for Condition and Sex variables were Overground and Male, respectively.

Table S2. Adjusted and unadjusted model estimates of the effects of condition, velocity, leg length, BMI, and sex on variability outcomes.

Outcome Measure	Model Type	Condition: Treadmill	Condition: Treadmill + Handrail	Velocity	Leg Length	BMI	Sex: Female
CV% Step Length	Adjusted	**25.884 (21.692, 30.214)**	**27.623 (23.893, 31.370)**	-3.020 (-7.468, 1.543)	2.073 (-11.906, 16.078)	-0.003 (-0.169, 0.151)	0.961 (-0.818, 2.673)
	Unadjusted	**26.436 (22.700, 30.337)**	**28.091 (24.720, 31.595)**	-2.361 (-6.228, 1.790)			
CV% Step Width	Adjusted	-2.255 (-23.713, 16.917)	-4.358 (-24.900, 14.080)	2.855 (-21.241, 24.598)	**-98.963 (-162.379, -37.534)**	-0.152 (-0.828, 0.530)	-8.507 (-16.894, 0.036)
	Unadjusted	1.922 (-16.504, 20.263)	-1.018 (-19.169, 16.761)	7.725 (-12.917, 28.395)			
CV% Cadence	Adjusted	3.461 (-0.891, 7.419)	0.314 (-3.511, 3.810)	-3.100 (-7.800, 1.129)	4.893 (-6.360, 16.011)	0.067 (-0.044, 0.177)	1.046 (-0.306, 2.385)
	Unadjusted	3.625 (-0.280, 7.415)	0.490 (-3.003, 3.899)	-2.895 (-7.012, 1.095)			
CV% Step Time	Adjusted	3.160 (-1.538, 7.439)	-0.158 (-4.309, 3.622)	-3.732 (-8.842, 0.8590	4.385 (-7.545, 16.278)	0.088 (0.035, 0.211)	0.994 (-0.464, 2.449)
	Unadjusted	3.345 (-0.860, 7.460)	0.031 (-3.761, 3.725)	-3.497 (-7.972, 0.858)			
CV% Stance Time	Adjusted	**3.590 (0.081, 6.815)**	0.432 (-2.719, 3.295)	-2.542 (-6.388, 0.926)	5.478 (-4.461, 15.240)	0.064 (-0.044, 0.174)	1.087 (-0.138, 2.315)
	Unadjusted	**3.800 (0.695, 6.852)**	0.653 (-2.182, 3.406)	-2.290 (-5.630, 0.939)			
CV% Swing Time	Adjusted	**3.987 (0.745, 7.217)**	1.336 (-1.517, 4.161)	-1.726 (-5.190, 1.670)	4.099 (-4.178, 12.386)	0.022 (-0.065, 0.109)	0.484 (-0.567, 1.535)
	Unadjusted	**3.739 (0.753, 6.711)**	1.150 (-1.516, 3.776)	-2.011 (-5.134, 1.052)			
CV% Double Limb Support	Adjusted	**4.142 (1.924, 6.371)**	1.550 (-0.466, 3.562)	0.403 (-2.038, 2.842)	4.878 (-1.589, 11.297)	0.027 (-0.040, 0.095)	**0.888 (0.112, 1.661)**
	Unadjusted	**4.183 (2.067, 6.267)**	1.613 (-0.335, 3.530)	0.462 (-1.823, 2.714)			

Results reported as model estimate and 95% confidence interval. Significant terms are shown in bold ($p < 0.05$). Reference categories for Condition and Sex variables were Overground and Male, respectively.

Table S3. Adjusted models estimates of the effects of condition, velocity, leg length, BMI, and sex on turn outcomes.

		Condition		Leg Length	BMI	Sex
**Outcome Measure**	**Model Type**	Treadmill	Treadmill + Handrail			Female
**180° Turns**						
Duration (s)	Adjusted	**4.056 (3.703, 4.407)**	**3.095 (2.712, 3.474)**	-1.070 (-4.099, 1.558)	-0.013 (-0.039, 0.013)	**-0.320 (-0.650, -0.006)**
	Unadjusted	**4.061 (3.711, 4.412)**	**3.103 (2.714, 3.483)**			
Number of Steps	Adjusted	**5.657 (4.877, 6.437)**	**3.570 (2.960, 4.210)**	-4.156 (-11.127, 2.528)	0.022 (-0.040, 0.087)	-0.514 (-1.264, 0.233)
	Unadjusted	**5.671 (4.911, 6.433)**	**3.563 (2.930, 4.232)**			
Average Velocity (deg/s)	Adjusted	**-34.075 (-36.124, -32.014)**	**-30.128 (-32.694, -27.422)**	2.663 (-20.414, 25.758)	0.123 (-0.083, 0.328)	1.948 (-0.588, 4.497)
	Unadjusted	**-34.098 (-36.164, -32.039)**	**-30.251 (-32.828, -27.648)**			
Max Velocity (deg/s)	Adjusted	**-34.737 (-37.665, -31.789)**	**-31.039 (-34.194, -27.807)**	14.198 (-17.175, 48.192)	0.107 (-0.224, 0.436)	2.849 (-0.936, 6.619)
	Unadjusted	**-34.768 (-37.731, -31.822)**	**-31.010 (-34.152, -27.768)**			
**360° Turns**						
Duration (s)	Adjusted	**6.261 (5.730, 6.794)**	**4.681 (4.145, 5.235)**	-1.125 (-7.059, 4.883)	-0.002 (-0.079, 0.076)	-0.473 (-1.289, 0.339)
	Unadjusted	**6.261 (5.729, 6.791)**	**4.677 (4.144, 5.220)**			
Number of Steps	Adjusted	**9.732 (8.482, 10.973)**	**5.485 (4.587, 6.297)**	-3.326 (-13.508, 6.191)	0.009 (-0.090, 0.110)	-0.398 (-1.685, 0.931)
	Unadjusted	**9.734 (8.499, 10.986)**	**5.474 (4.586, 6.301)**			
Average Velocity (deg/s)	Adjusted	**-50.436 (-55.384, -45.556)**	**-44.541 (-48.385, -40.593)**	-1.437 (-35.023, 31.995)	-0.305 (-0.709, 0.105)	0.487 (-4.286, 5.272)
	Unadjusted	**-50.416 (-55.295, -45.548)**	**-44.559 (-48.411, -40.679)**			
Max Velocity (deg/s)	Adjusted	**-55.630 (-62.533, -48.821)**	**-49.283 (-54.112, -44.327)**	-17.094 (-81.926, 47.628)	-0.346 (-1.120, 0.435)	-0.623 (-9.908, 8.724)
	Unadjusted	**-55.630 (-62.470, -48.807)**	**-49.293 (-54.120, -44.392)**			

Results reported as model estimate and 95% confidence interval. Significant terms are shown in bold ($p < 0.05$). Reference categories for Condition and Sex variables were Overground and Male, respectively.
